# Synthesis and Characterization of 40 wt % Ce_0.9_Pr_0.1_O_2–*δ*_–60 wt % Nd_*x*_Sr_1−*x*_Fe_0.9_Cu_0.1_O_3−*δ*_ Dual-Phase Membranes for Efficient Oxygen Separation

**DOI:** 10.3390/membranes10080183

**Published:** 2020-08-12

**Authors:** Guoxing Chen, Zhijun Zhao, Marc Widenmeyer, Ruijuan Yan, Ling Wang, Armin Feldhoff, Anke Weidenkaff

**Affiliations:** 1 Department of Materials and Earth Sciences, Technische Universität Darmstadt, Alarich-Weiss-Str. 2, 64287 Darmstadt, Germany; marc.widenmeyer@mr.tu-darmstadt.de (M.W.); ruijuan.yan@mr.tu-darmstadt.de (R.Y.); lingwang0729@gmail.com (L.W.); 2 Institute of Physical Chemistry and Electrochemistry, Leibniz University Hannover, Callinstr. 3A, 30167 Hannover, Germany; zhijun.zhao@pci.uni-hannover.de (Z.Z.); armin.feldhoff@pci.uni-hannover.de (A.F.); 3 Fraunhofer Research Institution for Materials Recycling and Resource Strategies IWKS, Brentanostraße 2a, 63755 Alzenau, Germany

**Keywords:** oxygen separation, dual-phase membrane, CO_2_ tolerance, long-term stability, regenerative ability

## Abstract

Dense, H_2_- and CO_2_-resistant, oxygen-permeable 40 wt % Ce_0.9_Pr_0.1_O_2–_*_δ_*–60 wt % Nd*_x_*Sr_1−*x*_Fe_0.9_Cu_0.1_O_3−_*_δ_*dual-phase membranes were prepared in a one-pot process. These Nd-containing dual-phase membranes have up to 60% lower material costs than many classically used dual-phase materials. The Ce_0.9_Pr_0.1_O_2−_*_δ_*–Nd_0.5_Sr_0.5_Fe_0.9_Cu_0.1_O_3−_*_δ_* sample demonstrates outstanding activity and a regenerative ability in the presence of different atmospheres, especially in a reducing atmosphere and pure CO_2_ atmosphere in comparison with all investigated samples. The oxygen permeation fluxes across a Ce_0.9_Pr_0.1_O_2−_*_δ_*–Nd_0.5_Sr_0.5_Fe_0.9_Cu_0.1_O_3−_*_δ_* membrane reached up to 1.02 mL min^−1^ cm^−2^ and 0.63 mL min^−1^ cm^−2^ under an air/He and air/CO_2_ gradient at *T* = 1223 K, respectively. In addition, a Ce_0.9_Pr_0.1_O_2–_*_δ_*–Nd_0.5_Sr_0.5_Fe_0.9_Cu_0.1_O_3–_*_δ_* membrane (0.65 mm thickness) shows excellent long-term self-healing stability for 125 h. The repeated membrane fabrication delivered oxygen permeation fluxes had a deviation of less than 5%. These results indicate that this highly renewable dual-phase membrane is a potential candidate for long lifetime, high temperature gas separation applications and coupled reaction–separation processes.

## 1. Introduction

The change from a fossil fuel-based to a renewable energy-based system requires the development of resource efficient materials with a long lifetime for energy conversion technologies. In this regard, mixed ionic–electronic conducting (MIEC) oxygen transport materials have drawn increasing interest due to their high potential for various energy conversion applications such as the oxygen transport membrane (OTM) for producing oxygen from air [1,2,3,4,5,6,7,8,9,10,11,12], electrolytes for batteries [13,14,15], cathode materials for solid oxide fuel cells [16,17,18], catalysts [19,20,21], and in membrane reactors [21,22,23]. With the current background of increases in CO_2_ emissions and fossil resources depletion, CO_2_ capture and utilization have been intensively researched to reduce CO_2_ emissions, including thermolysis, photocatalysis, plasma, and electrochemical methods [24]. OTMs, processed in integrated oxy-fuel combustions, provide the oxygen supply and have been considered to be a highly efficient and cost-effective way of capturing CO_2_. Using the OTMs membrane technology, the costs and energy consumption for oxygen production could be reduced at least by 35% compared with the conventional cryogenic distillation method [25,26]. Besides the application of the separation of oxygen from air, MIEC membranes used in membrane reactors are getting increasing attention to couple the reaction and separation processes to save energy and simplify the process [21,22,23,27,28]. Plasma technologies have attracted increasing attention as a timely flexible method for CO_2_ conversion and utilization [29,30,31,32,33]. For example, plasma-assisted MIEC membranes, used to separate oxygen radicals without additional external heating from the decomposition products, provide an efficient way to enhance the overall CO_2_ conversion efficiency via inhibiting the reverse reactions [27,28]. Traditional OTMs, which have been intensively investigated in recent years, are usually Ba^2+^- or Sr^2+^-containing perovskite-type oxides, such as Ba_1−*x*_Sr*_x_*Co_1−*y*_Fe*_y_*O_3−_*_δ_*, which show excellent oxygen permeabilities [4,5,34,35,36,37,38,39]. However, due to their vulnerability in CO_2_-containing environments, the widespread industrial applicability is strongly limited [1,4]. Although many studies have been devoted to modify perovskite-type OTMs to improve CO_2_ resistance with an appropriate selection of *A*- and *B*-site cations [40,41,42,43,44,45], most of such perovskite-type membranes are still susceptible to the formation of carbonates in the presence of CO_2_ due to unfavorable thermodynamics [15].

Dual-phase membranes consisting of mechanically and chemically robust fluorite oxides as an oxygen ionic conducting phase and a Ruddlesden–Popper, perovskite, or spinel oxide as an electronic conducting phase, are promising alternatives to ensure chemical and mechanical stability and a high oxygen permeability. For choosing the ionic conducting materials, three criteria should be fulfilled as discussed by Zhu et al. [2]: (i) high ionic conductivity; (ii) high chemical resistance under reducing or acidic (such as CO_2_) gas atmospheres; (iii) good chemical compatibility with the selected electronic conducting materials. Ceria-based materials with a fluorite structure fulfil these three criteria and have been well-established as ionic conductor phases for oxide anions in dual-phase membranes. Besides, the formation of continuous ionic and electronic conducting paths in dual-phase membranes is needed. To ensure this, MIEC oxides (such as *Ln_x_*Sr_1−*x*_FeO_3−*δ*_ *Ln* = lanthanides) providing both electronic and oxygen ionic transport paths are often considered as promising electronic conducting materials [2,3,4,6,46,47,48,49,50,51,52]. Similar selection criteria for the ionic conducting material have to be considered allowing for high electronic conductivity in combination with a high chemical and thermal compatibility [4]. The above-mentioned factors have made Ce_0.8_Sm_0.2_O_2−_*_δ_*–Sm_0.5_Sr_0.5_Fe_0.8_Cu_0.2_O_3−_*_δ_* (CSO-SSFCO), Ce_0.9_Pr_0.1_O_2−_*_δ_*–Pr_0.6_Ca_0.4_FeO_3−_*_δ_* (CPO-PCFO), Ce_0.8_Gd_0.2_O_2−_*_δ_*–Pr_0.5_Sr_0.5_Fe_0.5_Co_0.5_O_3−_*_δ_* (CGO-PSFCO), and Ce_0.9_Nd_0.1_O_2−_*_δ_*–Nd_0.6_Sr_0.4_CoO_3−_*_δ_* (CNO-NSCO) intensively investigated materials [2,3,4,6,46,47,48,49,50,51,52,53,54,55,56,57,58,59,60,61,62,63,64,65,66,67]. Although the CO_2_ resistance has been improved, most of their oxygen permeation fluxes are still too low to meet the requirements for practical applications. Therefore, OTMs which combine good chemical stability in different varying atmospheres with a high oxygen permeability are still highly needed for long lifetime industrial applications. Omar et al. found that Nd-doped ceria exhibits the highest ionic conductivity among 10 mol% rare earth (Gd, Er, Lu, Dy, Yb, Y, Sm, Sm/Nd, Nd)-doped ceria systems prepared under similar conditions [68]. Based on these findings, a Nd-containing dual-phase membrane with the composition 60 wt % Ce_0.9_Nd_0.1_O_2−_*_δ_*–40 wt % Nd_0.6_Sr_0.4_FeO_3−_*_δ_* has been developed by Luo et al. [49]. A high oxygen permeation flux of 0.48 mL min^−1^ cm^−2^ was obtained for the membrane (0.6 mm thickness) with a porous La_0.6_Sr_0.4_CoO_3−*δ*_ coating layer using CO_2_ as sweep gas at 1123 K. In a recent study, the same authors designed and synthesized another novel Nd-containing dual-phase membrane (Ce_0.9_Nd_0.1_O_2−_*_δ_*–Nd_0.6_Sr_0.4_CoO_3−_*_δ_*), and the oxygen permeation flux was improved to 0.55 mL min^−1^ cm^−2^ through a membrane (0.6 mm thickness) under an air/CO_2_ gradient at 1223 K [50]. However, the oxygen permeation flux is still lower than other reported dual-phase membranes but still competitive in particular if the up to 60% lower materials cost are considered. In addition, the reproducibility and level of regeneration of the dual-phase membranes have received less attention in the literature for the oxygen transport process. In our previous studies on dual-phase membranes, different Ce_0.9_Pr_0.1_O_2−*δ*_:La_0.5_Sr_0.5_Fe_0.9_Cu_0.1_O_3−*δ*_ weight ratios (20%:80%, 40%:60%, 60%:40%, and 80%:20%,) were presented, with the highest permeation fluxes under an air/He and an air/CO_2_ gradient found for a weight ratio of 40%:60% [6]. Thus, inspired by the reported studies and our previous studies [6,49,50,68], here we report the CO_2_-resistant dual-phase 40 wt % Ce_0.9_Pr_0.1_O_2−_*_δ_*–60 wt % Nd*_x_*Sr_1−*x*_Fe_0.9_Cu_0.1_O_3−_*_δ_* (*x* = 0.2, 0.5, 0.8) membranes prepared by a one-pot synthesis. This approach offers the possibility to upscale the production by a continuous spray combustion aerosol process for industrial applications [69]. The desired structural and compositional stability or level of regeneration and the simultaneous continuous oxygen permeability under air/CO_2_ and air/He gradients of these dual-phase membranes are investigated in view of an enhanced lifetime for a robust active system. The dual-phase membranes are tested towards CO_2_ at different temperatures and times in order to study the chemical resistance under a CO_2_ atmosphere. Long-term CO_2_ stability and oxygen permeability are monitored and evaluated. In addition, the reproducibility and level of regeneration of the dual-phase membranes have been confirmed for a power-to-x conversion application.

## 2. Experimental

### 2.1. Preparation of Powders and Membranes

CO_2_-tolerant 40 wt % Ce_0.9_Pr_0.1_O_2−_*_δ_*–60 wt % Nd*_x_*Sr_1−*x*_Fe_0.9_Cu_0.1_O_3−_*_δ_* (*x* = 0.2, 0.5, 0.8) dual-phase membranes were prepared by the same one-pot method as previously reported for Ce_0.9_Pr_0.1_O_2−*δ*_–La_0.5_Sr_0.5_Fe_0.9_Cu_0.1_O_3−*δ*_ [6] using Nd(NO_3_)_3_·6H_2_O (99.9%, Alfa Aesar, Kandel, Germany) instead of La(NO_3_)_3_·6H_2_O. Then, the as-prepared Ce_0.9_Pr_0.1_O_2−*δ*_–Nd*_x_*Sr_1–*x*_Fe_0.9_Cu_0.1_O_3−*δ*_ dual-phase powders were pressed with 60 kN (equals 40 MPa for a 16 mm diameter) to get the green disks. These green disks were sintered at 1323 K for 15 h in air (3 K min^−1^ heating and cooling rate). The desired membrane thickness (~0.6 mm) was adjusted by polishing with a 600 grit sandpaper. The thickness of the membranes used for oxygen permeation tests were 0.6 - 0.65 mm.

### 2.2. Materials Characterization

Powder X-ray diffraction (XRD, Mo-*Kα*_1_, a STOE STADI P X-ray diffractometer, Darmstadt, Germany) was used to identify the phase structures of the samples at room temperature. In situ XRD measurements were performed from 303 K to 1123 K in air (Bruker D8 Advance instrument with Mo-*Kα*_1_ radiation, Billerica, MA, USA). Thermogravimetric analysis (TGA) measurements were performed to characterize the weight changes of the as-prepared membranes (NETZSCH STA 449C, Selb, Germany) in different atmospheres (pure CO_2_, air, Ar or 5% H_2_-95% Ar). The powder samples were placed in a NETZSCH STA 449F3 (Selb, Germany) for various times and temperatures in order to investigate the chemical stability towards CO_2_. Scanning electron microscopy (SEM, Zeiss Gemini 500, Oberkochen, Germany) was used to study the microstructures of the prepared dual-phase membranes. The elemental distributions were investigated by energy-dispersive X-ray spectroscopy (EDXS) (Bruker XFlash 6|60, Billerica, MA, USA).

### 2.3. Oxygen Permeation Measurements

Oxygen permeability measurements were carried out on a home-made high-temperature permeation device shown in Figure S1. The disk membranes were sealed on an alumina tube (16 mm) with a gold paste (CHEMPUR, Karlsruhe, Germany). In this work, synthetic air was used as the feed gas and CO_2_ or He as the sweep gas. A detailed description of the setup and process parameters for oxygen permeation measurements can be found in our previous studies [6,40]. Concerning the oxygen permeation tests, four different 40 wt % Ce_0.9_Pr_0.1_O_2−_*_δ_*–60 wt % Nd_0.5_Sr_0.5_Fe_0.9_Cu_0.1_O_3−_*_δ_* membranes have been tested to investigate the reproducibility and level of regeneration. Partly due to the leakage of the gold sealing paste, a higher nitrogen concentration in the permeate gas was detected. The purity of the permeating O_2_ is around 65%~85% for different oxygen permeation tests. All oxygen permeation measurements were leakage corrected. 

## 3. Results and Discussion

### 3.1. Structure Characterization and Morphologies

**Figure 1:**
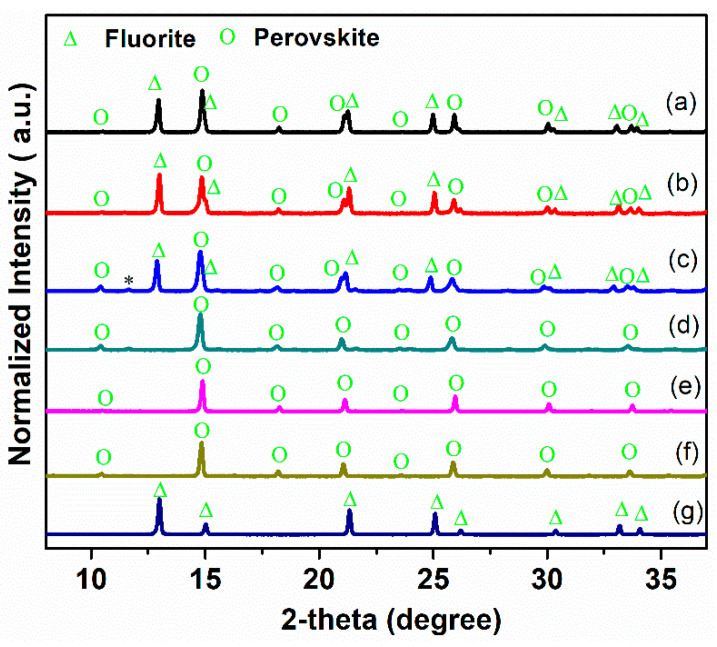
XRD patterns of Ce_0.9_Pr_0.1_O_2−_*_δ_*–Nd*_x_*Sr_1–*x*_Fe_0.9_Cu_0.1_O_3−_*_δ_* (*x* = 0.2, 0.5, 0.8) dual-phase membranes. (**a**) Ce_0.9_Pr_0.1_O_2−_*_δ_*–Nd_0.2_Sr_0.8_Fe_0.9_Cu_0.1_O_3−_*_δ_*; (**b**) Ce_0.9_Pr_0.1_O_2−_*_δ_*–Nd_0.5_Sr_0.5_Fe_0.9_Cu_0.1_O_3−_*_δ_*; (**c**) Ce_0.9_Pr_0.1_O_2−_*_δ_*–Nd_0.8_Sr_0.2_Fe_0.9_Cub;;_0.1_O_3−_*_δ_*; (**d**) Nd_0.2_Sr_0.8_Fe_0.9_Cu_0.1_O_3−_*_δ_*; (**e**) Nd_0.5_Sr_0.5_Fe_0.9_Cu_0.1_O_3−_*_δ_*; (**f**) Nd_0.8_Sr_0.2_Fe_0.9_Cu_0.1_O_3−_*_δ_*; (g) Ce_0.9_Pr_0.1_O_2−_*_δ_*. *: impurity reflection.

The XRD patterns of the Ce_0.9_Pr_0.1_O_2−_*_δ_*–Nd*_x_*Sr_1−*x*_Fe_0.9_Cu_0.1_O_3−_*_δ_* (*x* = 0.2, 0.5, 0.8) powders and the reference samples (Ce_0.9_Pr_0.1_O_2−_*_δ_* and Nd*_x_*Sr_1–*x*_Fe_0.9_Cu_0.1_O_3−_*_δ_*) are shown in Figure 1. Ce_0.9_Pr_0.1_O_2−_*_δ_*–Nd_0.2_Sr_0.8_Fe_0.9_Cu_0.1_O_3−_*_δ_* and Ce_0.9_Pr_0.1_O_2−_*_δ_*–Nd_0.5_Sr_0.5_Fe_0.9_Cu_0.1_O_3−_*_δ_* composite materials are composed of a fluorite-type phase and a perovskite-type phase, indicating a sufficient chemical compatibility between Ce_0.9_Pr_0.1_O_2−_*_δ_* and Nd*_x_*Sr_1–*x*_Fe_0.9_Cu_0.1_O_3−_*_δ_*(*x* = 0.2 and *x* = 0.5). However, with a further increasing Nd content (*x* = 0.8), an additional weak reflection at 2*θ* = 11.8° points to the presence of an unidentified impurity phase, which suggests some reaction occurring, thus, a reduced chemical compatibility between Ce_0.9_Pr_0.1_O_2−_*_δ_* and Nd_0.8_Sr_0.2_Fe_0.9_Cu_0.1_O_3−_*_δ_*.

**Figure 2:**
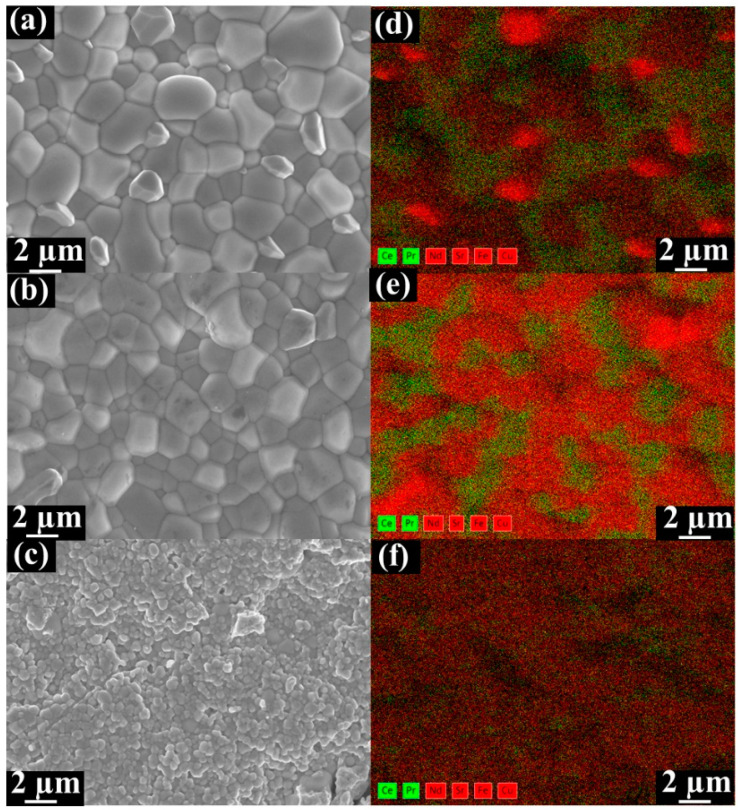
SEM and the corresponding energy-dispersive X-ray spectroscopy (EDXS) images of fresh unpolished Ce_0.9_Pr_0.1_O_2−_*_δ_*–Nd*_x_*Sr_1−*x*_Fe_0.9_Cu_0.1_O_3−_*_δ_* dual-phase membranes. (**a**) Ce_0.9_Pr_0.1_O_2−_*_δ_*–Nd_0.2_Sr_0.8_Fe_0.9_Cu_0.1_O_3−_*_δ_*; (**b**) Ce_0.9_Pr_0.1_O_2−_*_δ_*–Nd_0.5_Sr_0.5_Fe_0.9_Cu_0.1_O_3−_*_δ_*; (**c**) Ce_0.9_Pr_0.1_O_2−_*_δ_*–Nd_0.8_Sr_0.2_Fe_0.9_Cu_0.1_O_3−_*_δ_*. Contribution to the EDXS mapping data (**d**–**f**) resulting from the fluorite phases (Ce *Lα* and Pr *Lα*) are marked in green and in red for the perovskite-type phases (Nd *Lα*, Sr *Lα*, Fe *Kα* and Cu *Lα*).

SEM-EDXS analyses were conducted to investigate the feasibility of the dual-phase membranes. Figure 2 displays the SEM and EDXS micrographs of fresh Ce_0.9_Pr_0.1_O_2−_*_δ_*–Nd*_x_*Sr_1−*x*_Fe_0.9_Cu_0.1_O_3−_*_δ_* dual-phase membranes. The grains are closely packed without cracks for Ce_0.9_Pr_0.1_O_2−_*_δ_*–Nd_0.2_Sr_0.8_Fe_0.9_Cu_0.1_O_3−_*_δ_* and Ce_0.9_Pr_0.1_O_2−_*_δ_*–Nd_0.5_Sr_0.5_Fe_0.9_Cu_0.1_O_3−_*_δ_* samples. Ce_0.9_Pr_0.1_O_2−_*_δ_* and Nd*_x_*Sr_1_–*_x_*Fe_0.9_Cu_0.1_O_3−_*_δ_* grains can be clearly distinguished, as shown in Figure 2a,b, which indicates a good chemical compatibility of the two phases in the dual-phase membranes. The Ce_0.9_Pr_0.1_O_2−_*_δ_*–Nd_0.8_Sr_0.2_Fe_0.9_Cu_0.1_O_3−_*_δ_* sample reveals a smaller average grain diameter than Ce_0.9_Pr_0.1_O_2−_*_δ_*–Nd_0.2_Sr_0.8_Fe_0.9_Cu_0.1_O_3−_*_δ_* and Ce_0.9_Pr_0.1_O_2−_*_δ_*–Nd_0.5_Sr_0.5_Fe_0.9_Cu_0.1_O_3−_*_δ_* samples. In addition, small amounts of pinholes are found as shown in Figure 2c. These observations demonstrate that a suitable Nd content in the dual-phase membranes can form a good percolation network with well-separated oxygen ion conductor (Ce_0.9_Pr_0.1_O_2−_*_δ_*) and mixed ionic electronic conductor (Nd*_x_*Sr_1–*x*_Fe_0.9_Cu_0.1_O_3−_*_δ_*).

### 3.2. Materials Tolerance towards Air, CO_2_ and H_2_

**Figure 3:**
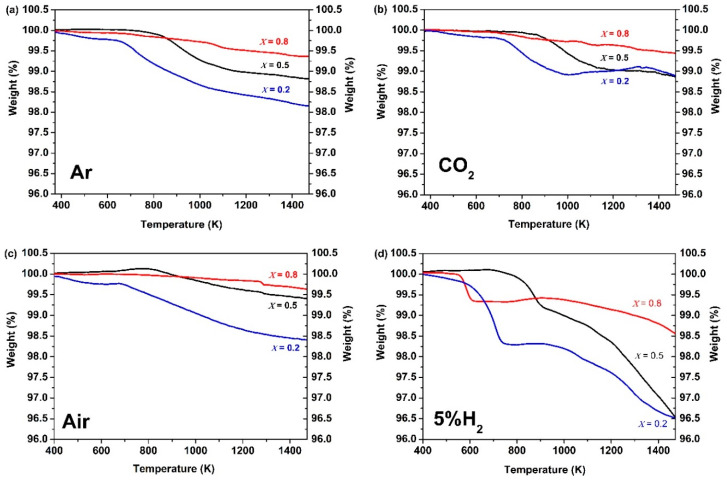
Thermogravimetric (TG) curves of Ce_0.9_Pr_0.1_O_2−_*_δ_*–Nd*_x_*Sr_1–*x*_Fe_0.9_Cu_0.1_O_3−_*_δ_* (*x* = 0.2, 0.5, 0.8) dual-phase membranes under flowing (**a**) Ar, (**b**) CO_2_, (**c**) air and (**d**) 5 vol% H_2_-95 vol% Ar atmospheres (heating rate: 10 K min^−1^).

**Figure 4:**
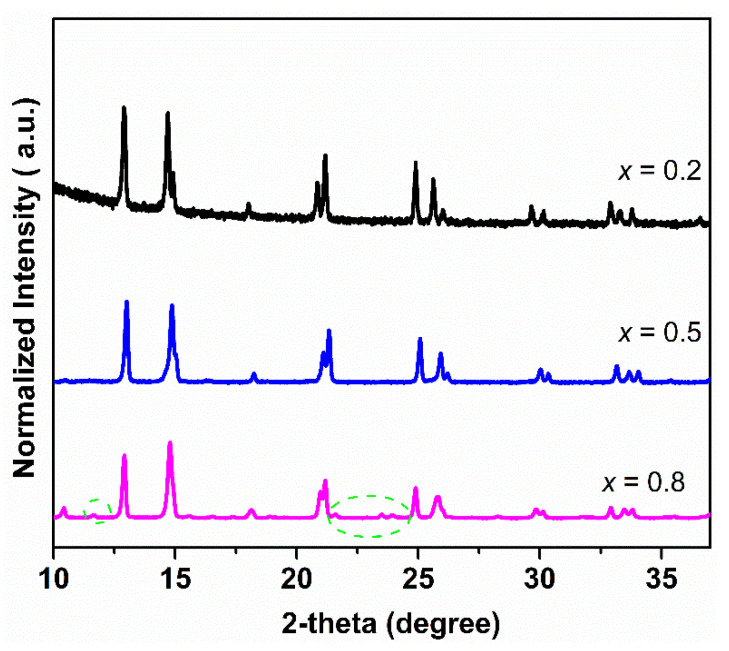
XRD patterns of Ce_0.9_Pr_0.1_O_2−_*_δ_*–Nd*_x_*Sr_1−*x*_Fe_0.9_Cu_0.1_O_3−_*_δ_* (*x* = 0.2, 0.5, 0.8) samples after TGA measurements were made under a CO_2_ atmosphere.

The TGA curves of Ce_0.9_Pr_0.1_O_2−_*_δ_*–Nd*_x_*Sr_1−*x*_Fe_0.9_Cu_0.1_O_3−_*_δ_* (*x* = 0.2, 0.5, 0.8) dual-phase membranes under air, CO_2_, Ar, and 5 vol% H_2_-95 vol% Ar atmospheres are shown in Figure 3, in order to determine the potential carbonate formation, CO_2_ adsorption ability, oxygen vacancy formation, as well as H_2_ tolerance of the membranes. The TGA curves of membrane materials reveal a comparable response to the changes of the gas atmosphere. The higher the reductive power (air < CO_2_ < Ar < Ar/H_2_) of the surrounding gas atmosphere, the larger the resulting mass change. A second general trend is that the higher the Sr content in Ce_0.9_Pr_0.1_O_2−_*_δ_*–Nd*_x_*Sr_1−*x*_Fe_0.9_Cu_0.1_O_3−_*_δ_* (*x* = 0.2, 0.5, 0.8) the easier, meaning it can be obtained at a lower temperature, and the stronger the occurring mass change. A similar trend of an increasing mass change has been reported in other studies for perovskite-type samples with higher contents of Sr^2+^ [36,70]. In contrast to Ar/H_2_ atmospheres, Ce_0.9_Pr_0.1_O_2−_*_δ_*–Nd*_x_*Sr_1−*x*_Fe_0.9_Cu_0.1_O_3−_*_δ_* revealed a more or less continuous mass change upon heating to 1473 K in air, CO_2_ and Ar. This points to a formation of oxygen vacancies by local thermal reduction. An exception is Ce_0.9_Pr_0.1_O_2−_*_δ_*–Nd_0.2_Sr_0.8_Fe_0.9_Cu_0.1_O_3−_*_δ_* since it shows a reversible mass change above 1000 K under CO_2_ exposure. This can be attributed to the higher Sr content and enlarged sensitivity towards the intermediate formation of SrCO_3_. As a result of the stronger CO_2_ adsorption ability of Ce_0.9_Pr_0.1_O_2−_*_δ_*–Nd_0.2_Sr_0.8_Fe_0.9_Cu_0.1_O_3−_*_δ_*, a smaller mass change is measured. For Ce_0.9_Pr_0.1_O_2−_*_δ_*–Nd*_x_*Sr_1–*x*_Fe_0.9_Cu_0.1_O_3−_*_δ_* (*x* = 0.5, 0.8), no indications of carbonate formation were visible. The very similar measurement values of the total mass changes at 1473 K as in Ar may suggest a certain adsorption of CO_2_ on the membrane surface and have to be further investigated (see below). A similar behavior was already reported in our previous study [40]. In the Ar/H_2_ atmosphere, a clearly different progression of the TGA curves was observed. In the temperature range 600 K ≤ *T* ≤ 800 K, a defined mass change was obtained resulting from a change in the perovskite phase. However, the exact origin is still unknown. This is around 900 K followed by a continuous mass change pointing again to a thermal reduction by oxygen vacancy formation. Another obvious feature of the TGA curves is that Ce_0.9_Pr_0.1_O_2−_*_δ_*–Nd_0.8_Sr_0.2_Fe_0.9_Cu_0.1_O_3−_*_δ_* showed the easiest reaction in the first step, while the thermal reduction at a higher temperature follows the trend as observed in the other gas atmospheres. This can be explained by the determined differences in the microstructure of the membrane materials, since Ce_0.9_Pr_0.1_O_2−_*_δ_*–Nd_0.8_Sr_0.2_Fe_0.9_Cu_0.1_O_3−_*_δ_* has a much smaller grain size (Figure 2) resulting in an easier access of H_2_ from the surrounding gas atmosphere. Figure 4 shows the XRD patterns of Ce_0.9_Pr_0.1_O_2−_*_δ_*–Nd*_x_*Sr_1−*x*_Fe_0.9_Cu_0.1_O_3−_*_δ_* (*x* = 0.2, 0.5, 0.8) samples after TGA measurements under a CO_2_ atmosphere. Evidently, no secondary phases and carbonates are found for Ce_0.9_Pr_0.1_O_2−_*_δ_*–Nd_0.5_Sr_0.5_Fe_0.9_Cu_0.1_O_3−_*_δ_*, while some additional unidentified impurity phases causing reflections at about 2*θ* = 11.5°, 21.2°, and 24° are formed for Ce_0.9_Pr_0.1_O_2−_*_δ_*–Nd_0.8_Sr_0.2_Fe_0.9_Cu_0.1_O_3−_*_δ_* and Ce_0.9_Pr_0.1_O_2−_*_δ_*–Nd_0.2_Sr_0.8_Fe_0.9_Cu_0.1_O_3−_*_δ_* samples. These results point to a good thermal and chemical compatibility under a CO_2_ atmosphere and can be achieved with a suitable Nd content in the dual-phase membranes.

**Figure 5:**
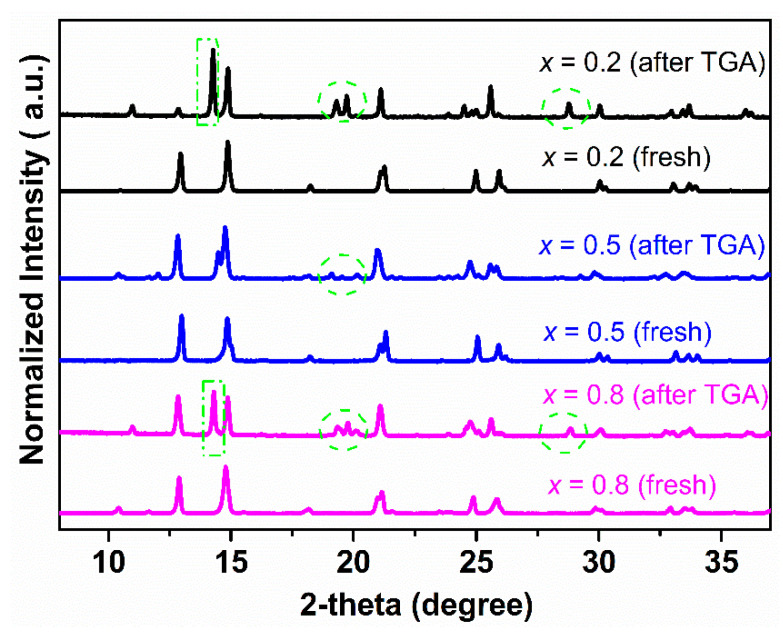
XRD patterns of Ce_0.9_Pr_0.1_O_2−_*_δ_*–Nd*_x_*Sr_1–*x*_Fe_0.9_Cu_0.1_O_3−_*_δ_* (*x* = 0.2, 0.5, 0.8) bulk samples before and after TGA measurements under a 5 vol% H_2_-95 vol% Ar atmosphere.

Figure 5 shows the XRD patterns of Ce_0.9_Pr_0.1_O_2−_*_δ_*–Nd*_x_*Sr_1−*x*_Fe_0.9_Cu_0.1_O_3−_*_δ_* (*x* = 0.2, 0.5, 0.8) bulk samples before and after TGA measurements under a 5 vol% H_2_-95 vol% Ar atmosphere (Figure 3d). Ce_0.9_Pr_0.1_O_2−_*_δ_*–Nd_0.5_Sr_0.5_Fe_0.9_Cu_0.1_O_3−_*_δ_* almost maintained its structure with the formation of a small fraction of unidentified impurity phases, while Ce_0.9_Pr_0.1_O_2−_*_δ_*–Nd_0.8_Sr_0.2_Fe_0.9_Cu_0.1_O_3−_*_δ_* and Ce_0.9_Pr_0.1_O_2−_*_δ_*–Nd_0.2_Sr_0.8_Fe_0.9_Cu_0.1_O_3−_*_δ_* were heavily destroyed by large fractions of impurity phases. This demonstrates that Ce_0.9_Pr_0.1_O_2−_*_δ_*–Nd_0.5_Sr_0.5_Fe_0.9_Cu_0.1_O_3−_*_δ_* has the largest resistance against reducing conditions.

**Figure 6:**
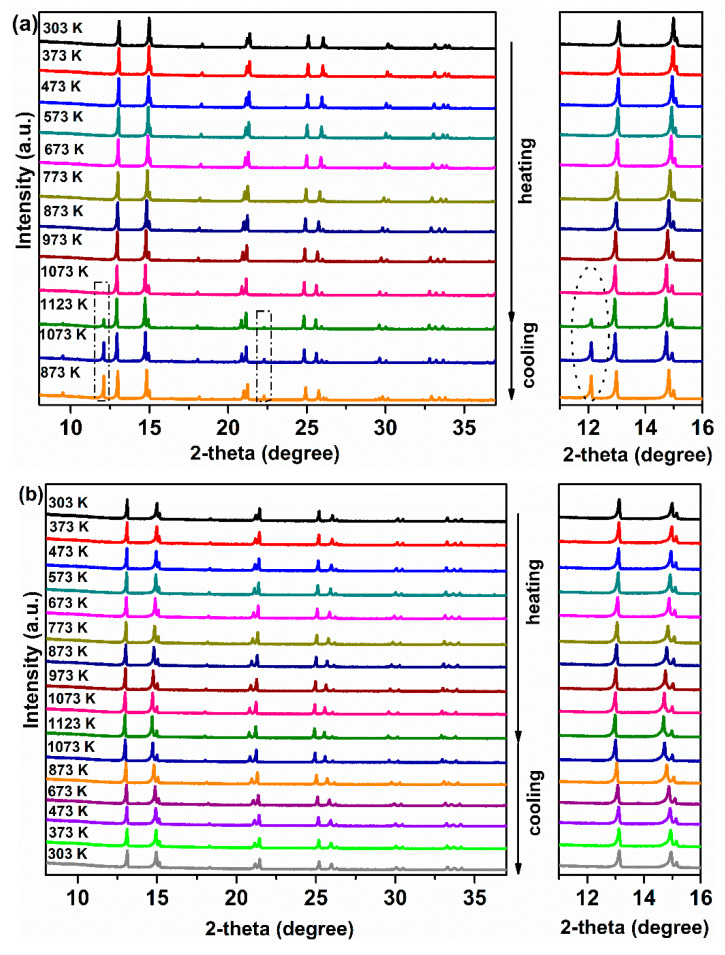

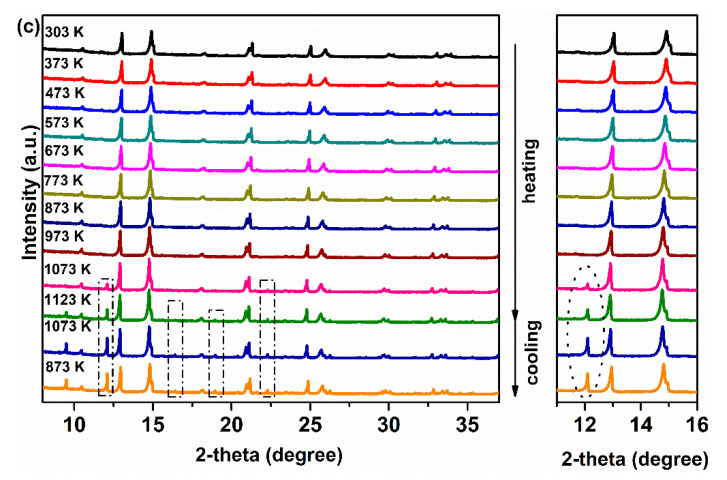
In situ XRD patterns of Ce_0.9_Pr_0.1_O_2−_*_δ_*–Nd*_x_*Sr_1−*x*_Fe_0.9_Cu_0.1_O_3−_*_δ_* powders in air: (**a**) Ce_0.9_Pr_0.1_O_2–_*_δ_*–Nd_0.2_Sr_0.8_Fe_0.9_Cu_0.1_O_3−_*_δ_*; (**b**) Ce_0.9_Pr_0.1_O_2−_*_δ_*–Nd_0.5_Sr_0.5_Fe_0.9_Cu_0.1_O_3−_*_δ_*; (**c**) Ce_0.9_Pr_0.1_O_2−_*_δ_*–Nd_0.8_Sr_0.2_Fe_0.9_Cu_0.1_O_3−_*_δ_*.

To study the phase stability of the membrane materials, Ce_0.9_Pr_0.1_O_2−_*_δ_*–Nd*_x_*Sr_1−*x*_Fe_0.9_Cu_0.1_O_3−_*_δ_* (*x* = 0.2, 0.5, 0.8) powders were evaluated by in situ XRD measurements in air. As displayed in Figure 6a, some additional reflections appear when the temperature goes up to 1123 K and still can be observed even after cooling down to 873 K for sample Ce_0.9_Pr_0.1_O_2−_*_δ_*–Nd_0.2_Sr_0.8_Fe_0.9_Cu_0.1_O_3−_*_δ_*. Similarly, for sample Ce_0.9_Pr_0.1_O_2−_*_δ_*–Nd_0.8_Sr_0.2_Fe_0.9_Cu_0.1_O_3−_*_δ_* (Figure 6c), some unidentified impurity phases were formed when the temperature goes up to 1073 K and still can be found even after cooling down to 873 K. Interestingly, as shown in Figure 6b for sample Ce_0.9_Pr_0.1_O_2−_*_δ_*–Nd_0.5_Sr_0.5_Fe_0.9_Cu_0.1_O_3−_*_δ_*, no structural phase transition and additional reflections were observed in the whole temperature range during heating and cooling in air. Both the perovskite-type phase and the fluorite-type phase remain unchanged. Thermal expansion coefficients of Ce_0.9_Pr_0.1_O_2−*δ*_ and Nd_0.5_Sr_0.5_Fe_0.9_Cu_0.1_O_3−*δ*_ are 7.27(3) × 10^−5^ K^−1^ and 5.30(5) × 10^−5^ K^−1^, respectively, in the temperature range 295–1123 K based on the XRD patterns in Figure 6b. All these results indicate a good chemical compatibility of these two phases and good structural stability in air. 

**Figure 7:**
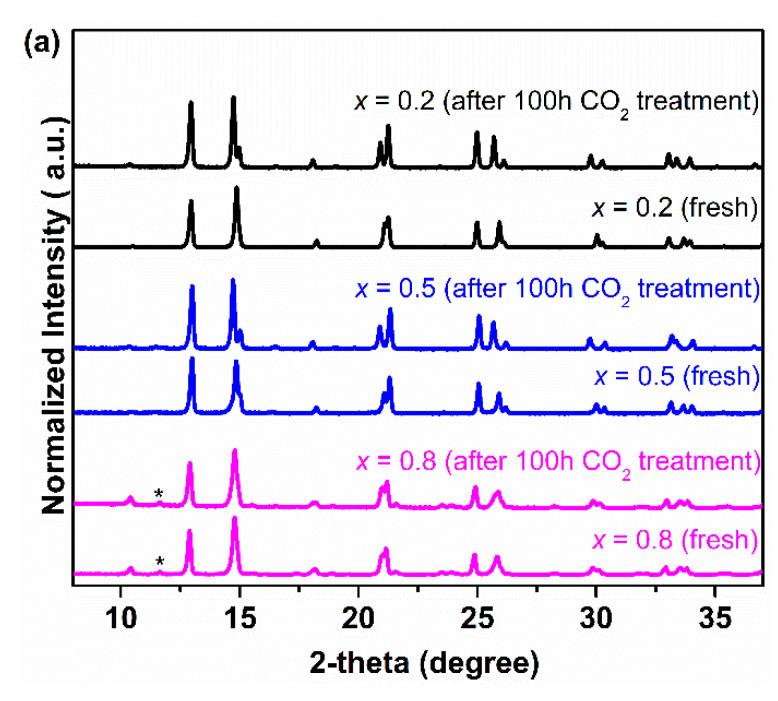

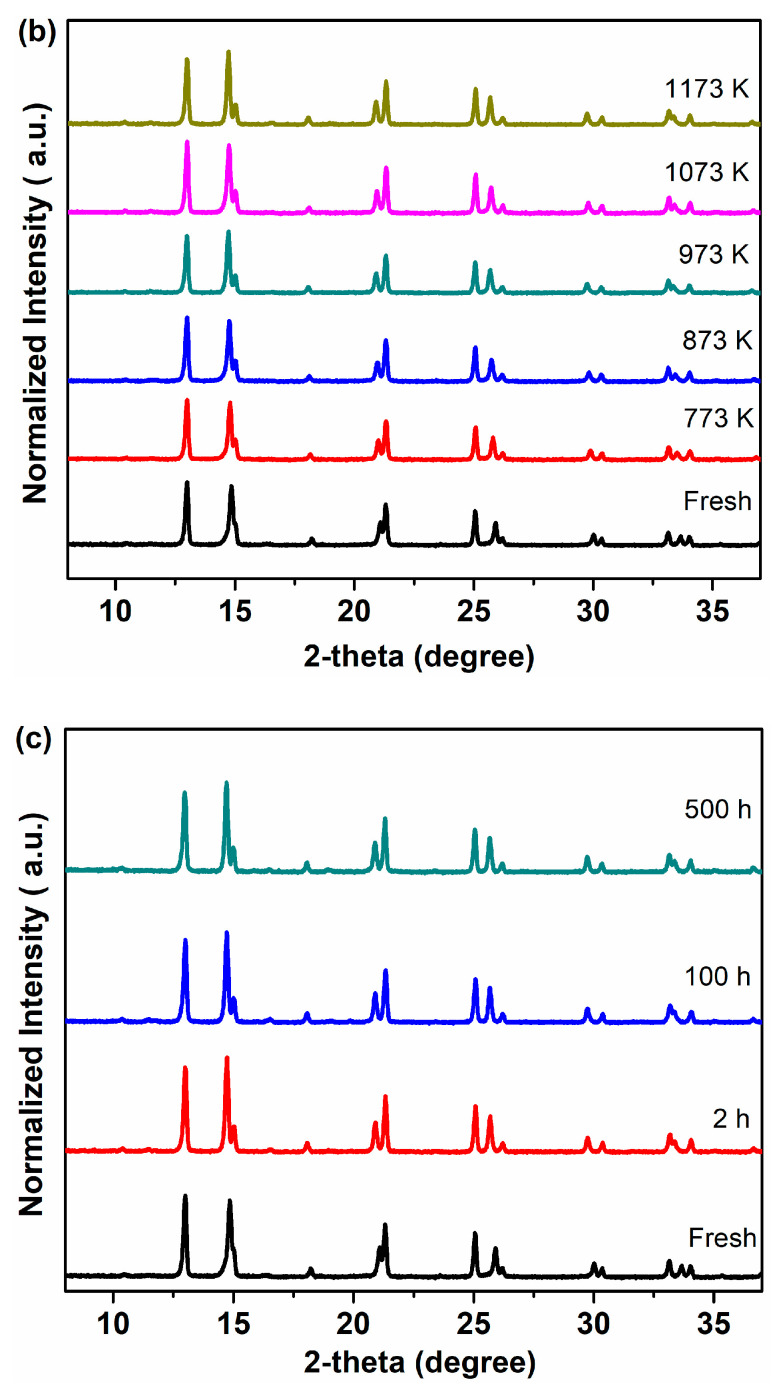
XRD patterns of the Ce_0.9_Pr_0.1_O_2−_*_δ_*–Nd*_x_*Sr_1−*x*_Fe_0.9_Cu_0.1_O_3−_*_δ_* (*x* = 0.2, 0.5, 0.8) powder samples after exposure to the CO_2_ atmosphere: (**a**) at 1173 K for 100 h; (**b**) Ce_0.9_Pr_0.1_O_2−_*_δ_*–Nd_0.5_Sr_0.5_Fe_0.9_Cu_0.1_O_3−_*_δ_* sample at different temperatures for 2 h; (**c**) Ce_0.9_Pr_0.1_O_2−_*_δ_*–Nd_0.5_Sr_0.5_Fe_0.9_Cu_0.1_O_3−_*_δ_* sample for different times at 1173 K.

In order to further study the CO_2_ tolerance of Ce_0.9_Pr_0.1_O_2−_*_δ_*–Nd*_x_*Sr_1–*x*_Fe_0.9_Cu_0.1_O_3−_*_δ_* (*x* = 0.2, 0.5, 0.8) membrane materials, several groups of experiments in the presence of CO_2_ were carried out and the results were given in Figure 7. Figure 7a shows the XRD patterns of all the samples after an exposure to CO_2_ at 1173 K for 100 h. It can be seen that no formation of carbonates and impurity phases happened for all the Nd-containing dual-phase samples. To gain a more accurate conclusion on the CO_2_ tolerance of Nd-containing dual-phase materials, Ce_0.9_Pr_0.1_O_2−_*_δ_*–Nd_0.5_Sr_0.5_Fe_0.9_Cu_0.1_O_3−_*_δ_* powder samples were treated in the presence of CO_2_ at different temperatures for 2 hours (773~1173 K) and the XRD patterns were displayed in Figure 7b. No formation of carbonates was found for the Ce_0.9_Pr_0.1_O_2−_*_δ_*–Nd_0.5_Sr_0.5_Fe_0.9_Cu_0.1_O_3−_*_δ_* samples at any temperatures comparing the XRD patterns of the pristine samples, which indicates a high chemical and thermal stability when in CO_2_ atmospheres. In addition, extending the exposing time to 100 h and 500 h as shown in Figure 7c, the Ce_0.9_Pr_0.1_O_2−_*_δ_*–Nd_0.5_Sr_0.5_Fe_0.9_Cu_0.1_O_3−_*_δ_* sample still kept its original structure.

### 3.3. Oxygen Permeation Measurements 

**Figure 8:**
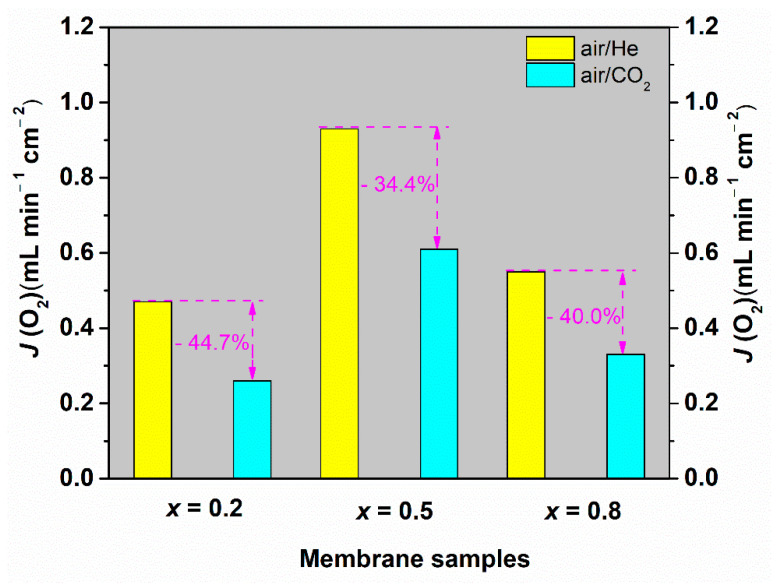
Oxygen permeation fluxes through the Ce_0.9_Pr_0.1_O_2−_*_δ_*–Nd*_x_*Sr_1−*x*_Fe_0.9_Cu_0.1_O_3−_*_δ_* (*x* = 0.2, 0.5, 0.8) membranes (thickness: 0.6 mm) under air/He and air/CO_2_ gradients at 1223 K.

**Figure 9:**
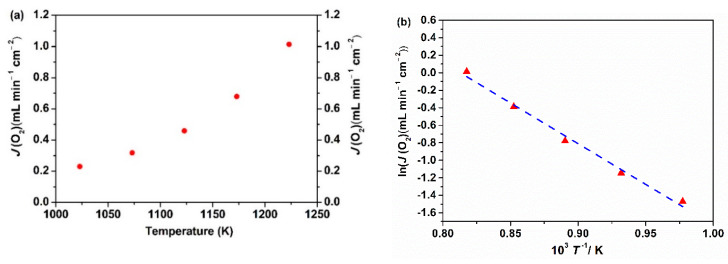
Oxygen permeation fluxes (**a**) and its Arrhenius plot (**b**) of the Ce_0.9_Pr_0.1_O_2−_*_δ_*–Nd_0.5_Sr_0.5_Fe_0.9_Cu_0.1_O_3−_*_δ_* dual-phase membrane (thickness: 0.6 mm) under an air/He gradient.

The oxygen permeation fluxes of the Ce_0.9_Pr_0.1_O_2–_*_δ_*–Nd*_x_*Sr_1−*x*_Fe_0.9_Cu_0.1_O_3−_*_δ_* (*x* = 0.2, 0.5, 0.8) membrane disks with a 0.6 mm thickness were measured at 1223 K under air/He and air/CO_2_ gradients. The oxygen permeation flux values of different membranes are based on the first 5 h oxygen permeation measurements at different gradients. As presented in Figure 8, the highest oxygen permeation flux of 0.94 mL min^−1^ cm^−2^ under an air/He gradient is obtained for the Ce_0.9_Pr_0.1_O_2−_*_δ_*–Nd_0.5_Sr_0.5_Fe_0.9_Cu_0.1_O_3−_*_δ_* membrane. When switching the sweep gas from He to CO_2_, the oxygen permeation rate declines to 0.61 mL min^−1^ cm^−2^. This decrease can be mainly attributed to chemical adsorption of CO_2_ on the oxygen vacancy sites, which strongly hampers the surface exchange reaction at the interface of lattice oxygen and oxygen vacancy. The strong influence of different gas atmospheres on the oxygen surface exchange rates was previously discussed in detail [5,71,72]. Besides, the variation of oxygen partial pressures on the permeate side of the membrane may also lead to a decrease in the oxygen permeation flux upon changing the sweep gas. A similar response has been found for the other two membranes. The high oxygen permeation flux through Ce_0.9_Pr_0.1_O_2−_*_δ_*–Nd_0.5_Sr_0.5_Fe_0.9_Cu_0.1_O_3−_*_δ_* can be attributed to the good percolation network of the Ce_0.9_Pr_0.1_O_2−_*_δ_* and Nd_0.5_Sr_0.5_Fe_0.9_Cu_0.1_O_3−_*_δ_* phases as well as the membrane phase stability at high temperatures as discussed in the previous sections. Given that the Ce_0.9_Pr_0.1_O_2−_*_δ_*–Nd_0.5_Sr_0.5_Fe_0.9_Cu_0.1_O_3−_*_δ_* dual-phase membrane shows the best performance, the oxygen permeation flux through the Ce_0.9_Pr_0.1_O_2−_*_δ_*–Nd_0.5_Sr_0.5_Fe_0.9_Cu_0.1_O_3−_*_δ_* dual-phase membrane (a new membrane) was measured in the temperature range of 1023 K < *T* < 1223 K under air/He gradients. As shown in Figure 9, the oxygen permeation fluxes of the membrane increased with temperature, showing a thermal activation behavior. Both bulk diffusion and surface oxygen exchange reaction processes are enhanced by higher temperatures. The oxygen permeation flux of this new Ce_0.9_Pr_0.1_O_2−_*_δ_*–Nd_0.5_Sr_0.5_Fe_0.9_Cu_0.1_O_3−_*_δ_* membrane reached 1.02 mL min^−1^ cm^−2^ at 1223 K under an air/He gradient. The deviation of the oxygen permeation flux with the previous value, as shown in Figure 8, is around 5% and reveals a promising reproducibility of the Ce_0.9_Pr_0.1_O_2−_*_δ_*–Nd_0.5_Sr_0.5_Fe_0.9_Cu_0.1_O_3−_*_δ_* dual-phase membranes. Slightly different surface morphologies and membrane thicknesses may result in the observed deviations. The activation energy of Ce_0.9_Pr_0.1_O_2−_*_δ_*–Nd_0.5_Sr_0.5_Fe_0.9_Cu_0.1_O_3−_*_δ_* dual-phase membrane is calculated to be 70 kJ mol^−1^, which is close to that of some other reported Nd-containing dual-phase membranes at similar conditions [49,50].

To gain insights into long-term CO_2_ durability and oxygen permeability, two additional Ce_0.9_Pr_0.1_O_2−_*_δ_*–Nd_0.5_Sr_0.5_Fe_0.9_Cu_0.1_O_3−_*_δ_* dual-phase membranes (thickness: 0.6 mm and 0.65 mm) were tested under air/He and air/CO_2_ gradients at 1223 K. As shown in Figure S2, the oxygen permeation flux through the 0.6 mm-thickness membrane reaches 0.97 mL min^−1^ cm^−2^ using He as the sweep gas, which has similar values as shown in Figure 8 and Figure 9. Switching He to CO_2_, the oxygen permeation rate goes down to 0.63 mL min^−1^ cm^−2^ and continues to decrease to a stable oxygen permeation flux around 0.3 mL min^−1^ cm^−2^ in the following 70 h period. 

**Figure 10:**
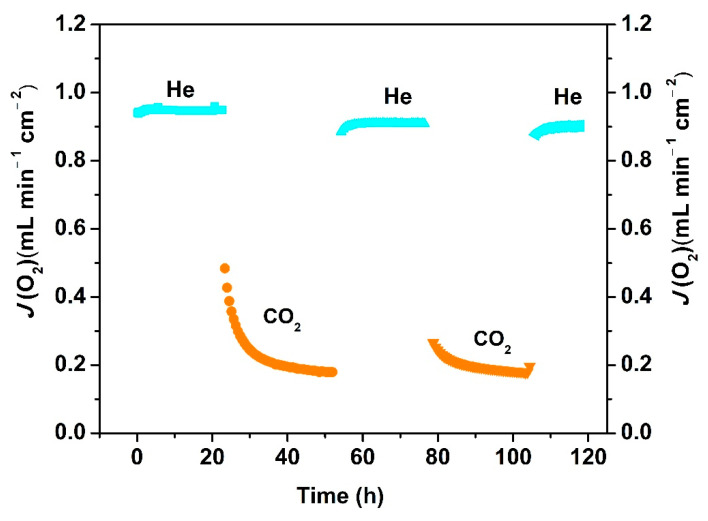
Long-term oxygen permeation flux through a Ce_0.9_Pr_0.1_O_2−_*_δ_*–Nd_0.5_Sr_0.5_Fe_0.9_Cu_0.1_O_3−_*_δ_* membrane at 1223 K under an air/He or air/CO_2_ gradient. Test conditions: 0.65 mm membrane thickness; 150 mL min^−1^ synthetic air as the feed gas; 29 mL min^−1^ CO_2_ as the sweep gas; 1 mL min^−1^ Ne as an internal standard gas.

**Figure 11:**
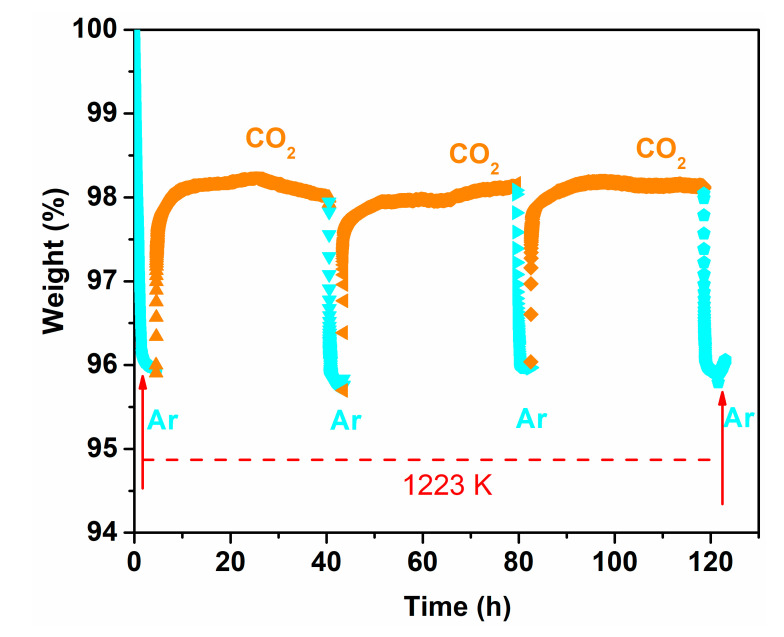
TG curve of the Ce_0.9_Pr_0.1_O_2−_*_δ_*–Nd_0.5_Sr_0.5_Fe_0.9_Cu_0.1_O_3−_*_δ_* dual-phase powder material while periodically changing the gas atmosphere between Ar and CO_2_ at 1223 K.

The reversibility of the oxygen permeability of the Ce_0.9_Pr_0.1_O_2−_*_δ_*–Nd_0.5_Sr_0.5_Fe_0.9_Cu_0.1_O_3−_*_δ_* dual-phase has been also evaluated. Figure 10 depicts the changeability of the oxygen permeation flux through the 0.65 mm-thick membrane as a function of time by periodically changing He or CO_2_ as the sweep gas at 1223 K. A high and stable oxygen permeation flux of 0.95 mL min^−1^ cm^−2^ was reached using He as the sweep gas in the first 20 h. Similar to the behaviors shown in Figure S2, the oxygen permeation decreases to 0.2 mL min^−1^ cm^−2^ in the following 30 h exposure to CO_2_. Upon switching the sweep gas back to He, the oxygen permeation flux can be quickly recovered to almost the initial oxygen flux value, as illustrated in Figure 10. After two CO_2_ and He gas cycles, the oxygen permeation flux can still be recovered to 0.92 mL min^−1^ cm^−2^. The reversibility of the oxygen permeation fluxes during such alternating gas exposures suggests that the decrease in the oxygen permeation process was dominated by the CO_2_ sorption on the Ce_0.9_Pr_0.1_O_2−_*_δ_*–Nd_0.5_Sr_0.5_Fe_0.9_Cu_0.1_O_3−_*_δ_* membrane surface, rather than the chemical reaction with CO_2_ forming carbonates on the membrane surface which generally leads to a complete loss of oxygen permeation flux. For instance, no permeation was reported for a BaCo_0.85_Bi_0.05_Zr_0.1_O_3−*δ*_ single phase membrane after using 10 vol% CO_2_-90 vol% He as a sweep gas due to the formation of BaCO_3_ [47]. No change of the crystal structures of the Ce_0.9_Pr_0.1_O_2−_*_δ_*–Nd_0.5_Sr_0.5_Fe_0.9_Cu_0.1_O_3−_*_δ_* dual-phase membrane is observed (as shown in Figure S3) before and after 70 h oxygen permeation under an air/CO_2_ gradient, revealing a high CO_2_ resistance. To further understand this behavior, the adsorption of CO_2_ on a Ce_0.9_Pr_0.1_O_2−_*_δ_*–Nd_0.5_Sr_0.5_Fe_0.9_Cu_0.1_O_3−_*_δ_* dual-phase membrane material was investigated experimentally by TGA when periodically changing the gas atmosphere between Ar and CO_2_ at 1223 K for 125 h. As clearly shown in Figure 11, the weight loss of Ce_0.9_Pr_0.1_O_2−_*_δ_*–Nd_0.5_Sr_0.5_Fe_0.9_Cu_0.1_O_3−_*_δ_* under CO_2_ (~2%) is significantly lower than that under the inert Ar gas (~4%) at 1223 K. When changing from Ar to CO_2_, a rapid weight gain to 98% of the initial weight is observed. During the CO_2_ exposure, a nearly constant weight was maintained. After switching back to Ar, a rapid weight loss to 96% occurred—observed after the first heating of Ar up to 1223 K. Additionally, the further switching of the gas atmosphere reveals a reproducible weight change between the above described values. All these observations suggest that the lattice oxygen releasing from the Ce_0.9_Pr_0.1_O_2–_*_δ_*–Nd_0.5_Sr_0.5_Fe_0.9_Cu_0.1_O_3–_*_δ_* membrane material is largely suppressed due to the strong CO_2_ adsorption or occupation of the oxygen vacancy sites by CO_2_. Generally, the surface exchange of oxygen and bulk diffusion of oxygen ions and electrons are the two rate-determining steps for the oxygen transport in MIEC membranes. From the reported data of various dual-phase membranes [50,65,66,67], the limitation of the oxygen transport process started to change from the oxygen bulk diffusion to surface oxygen exchange controlled when the thickness is less than 0.3 mm or 0.4 mm depending on the membrane material morphology, phase and operating conditions (gas atmosphere and temperature). Based on that, the bulk diffusion plays a dominant role in the oxygen permeation process through the 0.65 mm or 0.6 mm-thick membranes in this work. Decreasing the membrane thickness could be an effective way to further improve the oxygen permeation.

The oxygen permeabilitiesobtained within this work are compared with other dual-phase membranes under air/He and air/CO_2_ gradients in Table 1. Besides, the material costs for the representative membrane materials are also calculated, as shown in Table 1, based on the material costs of the used starting materials (metal source: nitrates) of dual-phase membrane materials summarized in Table S1. It can be seen that the Ce_0.9_Pr_0.1_O_2−_*_δ_*–Nd_0.5_Sr_0.5_Fe_0.9_Cu_0.1_O_3−_*_δ_* dual-phase membrane has a higher oxygen permeation flux (0.94 mL min^−1^ cm^−2^) compared to several types of dual-phase membranes under an air/He gradient in similar conditions. The oxygen permeation flux in this study is improved by up to 45% in comparison with the best reported value of other Nd-containing dual-phase membranes in the literature under an air/He gradient at similar conditions (see Table 1 below). The oxygen permeation flux under an air/CO_2_ gradient is comparable to that of other Nd-containing dual-phase membranes. The material cost of the prepared Nd-containing dual-phase membrane material in this work is 1.76 EUR/g. It can be seen that Nd-containing membrane materials are much cheaper compared to other membrane materials (Sm-, Gd-, and Pr-containing samples). Therefore, the newly developed regenerative Ce_0.9_Pr_0.1_O_2−_*_δ_*–Nd*_x_*Sr_1−*x*_Fe_0.9_Cu_0.1_O_3−_*_δ_* membrane with lower material costs have high potential for separating oxygen from H_2_-containing and high CO_2_-containing atmospheres, respectively.

## 4. Conclusions 

In this work, dense 40 wt % Ce_0.9_Pr_0.1_O_2−_*_δ_*–60 wt % Nd*_x_*Sr_1−*x*_Fe_0.9_Cu_0.1_O_3−_*_δ_* dual-phase membranes are synthesized by a scalable one-pot method. The characterization results (XRD and SEM-EDXS) reveal a matching chemical compatibility between the fluorite-type phase (Ce_0.9_Pr_0.1_O_2−_*_δ_*) and perovskite-type phase (Nd*_x_*Sr_1−*x*_Fe_0.9_Cu_0.1_O_3−_*_δ_*). The oxygen permeation flux under an air/He gradient is 45% higher than the best reported value of other Nd-containing dual-phase membranes in the literature at similar conditions. The oxygen permeation fluxes across a 0.6 mm thick Ce_0.9_Pr_0.1_O_2−_*_δ_*–Nd_0.5_Sr_0.5_Fe_0.9_Cu_0.1_O_3−_*_δ_* membrane reach up to 1.02 mL min^−1^ cm^−2^ under an air/He gradient at *T* = 1223 K. Besides, a Ce_0.9_Pr_0.1_O_2−_*_δ_*–Nd_0.5_Sr_0.5_Fe_0.9_Cu_0.1_O_3−_*_δ_* membrane (0.65 mm thickness) shows excellent long-term regenerative stability for 125 h. The significant poisoning effect of CO_2_ on the oxygen permeation fluxes through a Ce_0.9_Pr_0.1_O_2−_*_δ_*–Nd_0.5_Sr_0.5_Fe_0.9_Cu_0.1_O_3−_*_δ_* dual-phase membrane is mainly linked to the strong adsorption of CO_2_ to the membrane surface, which is experimentally verified by the TGA measurements with periodical changes of the gas atmosphere between Ar and CO_2_ at 1223 K for 125 h. The membranes show superior chemical resistance in the presence of CO_2_ and periodically regained their original structure for more than 500 h. In addition, the Ce_0.9_Pr_0.1_O_2−_*_δ_*–Nd_0.5_Sr_0.5_Fe_0.9_Cu_0.1_O_3−_*_δ_* membrane also demonstrates chemical durability in a H_2_-containing reducing atmosphere. Based on our findings, the newly developed Ce_0.9_Pr_0.1_O_2−_*_δ_*–Nd_0.5_Sr_0.5_Fe_0.9_Cu_0.1_O_3−_*_δ_* low-cost material appears to be a promising resource-efficient long lifetime membrane material for efficient high temperature oxygen transport membrane-based reactions owing to its structural stability in H_2_-containing and CO_2_ atmospheres together with a relatively high regenerative self-healing ability.

## Figures and Tables

**Table 1:** Summary of the reported promising oxygen permeation fluxes of various dual-phase membranes.

Sample	*J*(O_2_) (air/He) (mL min^−1^ cm^−2^)	*J*(O_2_) (air/CO_2_) (mL min^−1^ cm^−2^)	*d* (mm)	*T* (K)	*J*(O_2_) (air/CO_2_) Stability (h)	Material cost (EUR/g)	Ref.
CPO-NSFCO	0.97	0.32	0.6	1223	70	1.76	This work (Figure S2)
CPO-NSFCO	0.94	0.61	0.6	1223	5	1.76	This work (Figure 8)
CPO-NSFCO	1.02	--	0.6	1223	10	1.76	This work (Figure 9)
CPO-NSFCO	0.94	0.2	0.65	1223	125	1.76	This work (Figure 10)
CNO-NSFO	0.26	0.21	0.6	1223	120	1.70	[49]
CNO-NSCO	0.65	0.55	0.6	1223	150	1.89	[50]
CSO-SSCFO	1.01	0.7	0.6	1223	50	2.81	[52]
CPO-PSFCO	0.84	0.7	0.6	1223	400	2.23	[53]
CGO-PSCFO	0.6	0.45	0.5	1173	--	--	[55]
CGCO-LCFO	0.87	0.7	0.5	1223	--	--	[56]
LCO-30LSFO	0.32	0.19	0.6	1173	100	--	[59]
LCCO-30LSFO	0.45	0.27	0.6	1173	100	--	[59]
CSO-SCMCO	0.4	0.34	0.5	1173	75	--	[60]

CPO-NSFCO: 40wt % Ce_0.9_Pr_0.1_O_2−_*_δ_*–60wt % Nd0_0.5_Sr_0.5_Fe_0.9_Cu_0.1_O_3−_*_δ._* CNO-NSFO: 60 wt %Ce_0.9_ Nd_0.1_O_2−*δ*_–40 wt % Nd_0.6_Sr_0.4_FeO_3−*δ.*_ CNO-NSCO: 60 wt % Ce_0.9_Nd_0.1_O_2−*δ*_–40 wt % Nd_0.6_Sr_0.4_CoO_3−*δ.*_ CGO-PSCFO: 60 wt % Ce_0.8_Gd_0.2_O_2__–_*_δ_*– 40 wt % Pr_0.6_Sr_0.4_Co_0.5_Fe0_.5_O_3__–_*_δ._* CSO-SSCFO: 60 wt % Ce_0.8_Sm_0.2_O_2−_*_δ_*–40 wt % Sm_0.3_Sr_0.7_Cu_0.2_Fe_0.8_O_3−_*_δ._* CPO-PSFCO: 60 wt % Ce_0.9_Pr_0.1_O_2__–_*_δ_*–40 wt % Pr_0.6_Sr_0.4_Fe_0.5_Co_0.5_O_3__−_*_δ_*. LDCO-LSFO: 70 wt % La_0.15_Ce_0.85_O_2−*δ*–_30 wt % La_0.15_Sr_0.85_FeO_3−*δ.*_ CSO-SCMCO: 75wt % Ce_0.8_Sm_0.2_O_1.9–_25wt % Sm_0.8_Ca_0.2_Mn_0.5_Co_0.5_O_3_CGCO-LCFO: 75 wt % Ce_0.85_Gd_0.1_Cu_0.05_O_2−*δ*_–25 wt % La_0.6_Ca_0.4_FeO_3−*δ.*_ CPO-NSFCO: 40wt % Ce_0.9_Pr_0.1_O_2−_*_δ_*–60wt % Nd_0.5_Sr_0.5_Fe_0.9_Cu_0.1_O_3−_*_δ._* LCCO-LSFO: 70 wt % La_0.15_Ce_0.8_Cu_0.05_O_2−*δ*_ –30 wt % La_0.15_Sr_0.85_FeO_3−*δ.*_

## Supplementary Materials

The following are available online at https://www.mdpi.com/2077-0375/10/8/183/s1, Figure S1: Schematic diagram of the experimental setup for oxygen permeation measurements; Figure S2: Long-term oxygen permeation flux through a Ce_0.9_Pr_0.1_O_2−_*_δ_*–Nd_0.5_Sr_0.5_Fe_0.9_Cu_0.1_O_3−_*_δ_* membrane at 1223 K under air/He or air/CO_2_ gradient. Test conditions: 150 mL min−1 synthetic air as the feed gas; 29 mL min^−1^ CO_2_ as the sweep gas; 1 mL min^−1^ Ne as an internal standard gas; membrane thickness: 0.6 mm; Figure S3: XRD pattern of the sweep side (pure CO_2_) of the Ce_0.9_Pr_0.1_O_2−_*_δ_*–Nd_0.5_Sr_0.5_Fe_0.9_Cu_0.1_O_3−_*_δ_* dual-phase membrane after 70 h O_2_ permeation measurements as shown in Figure S2 under air/CO_2_ gradient (Bruker D8 Advance instrument with Cu-*K**α*_1,2_ radiation); Table S1: Summary of the starting material costs generally used in dual-phase membrane materials.
