# Supplementary material for: Synthesis and Characterization of 40 wt % Ce0.9Pr0.1O2–δ–60 wt % NdxSr1−xFe0.9Cu0.1O3−δ Dual-Phase Membranes for Efficient Oxygen Separation
Source: Membranes (Basel). 2020 Aug 12;10(8):183. doi: 10.3390/membranes10080183 (PMC7464960; doi:10.3390/membranes10080183)
Supplement: Supplementary file 1 [file membranes-10-00183-s001.pdf]

membranes

Supplementary Materials: Synthesis and Characterization of 40 wt% $Ce_{0.9}Pr_{0.1}O_{2-\delta}$–60 wt% $Nd_xSr_{1-x}Fe_{0.9}Cu_{0.1}O_{3-\delta}$ Dual-Phase Membranes for Efficient Oxygen Separation

Guoxing Chen [1,*], Zhijun Zhao [2], Marc Widenmeyer [1], Ruijuan Yan [1], Ling Wang [1], Armin Feldhoff [2] and Anke Weidenkaff [1,3,*]

1 Department of Materials and Earth Sciences, Technische Universität Darmstadt, Alarich-Weiss-Str. 2, 64287 Darmstadt, Germany, marc.widenmeyer@mr.tu-darmstadt.de (M.W.); ruijuan.yan@mr.tu-darmstadt.de (R.Y.); lingwang0729@gmail.com (L.W.)
2 Institute of Physical Chemistry and Electrochemistry, Leibniz University Hannover, Callinstr. 3A, 30167, Hannover, Germany; zhijun.zhao@pci.uni-hannover.de (Z.Z.); armin.feldhoff@pci.uni-hannover.de (A.F.)
3 Fraunhofer Research Institution for Materials Recycling and Resource Strategies IWKS, Rodenbacher Chaussee 4, 63457 Hanau, Germany
* Correspondence: guoxing.chen@mr.tu-darmstadt.de (G.C.); Anke.weidenkaff@mr.tu-darmstadt.de (A.W.)

Received: 27 July 2020; Accepted: 10 August 2020; Published: date

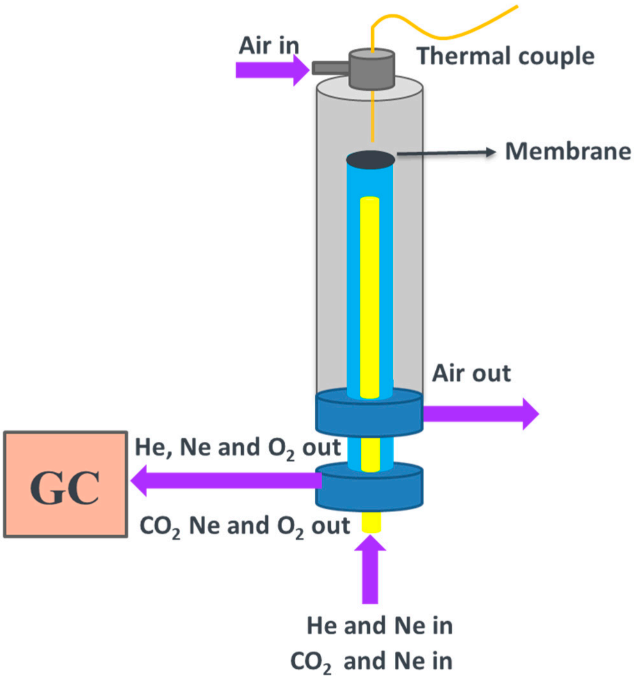

Figure S1. Schematic diagram of the experimental setup for oxygen permeation measurements.

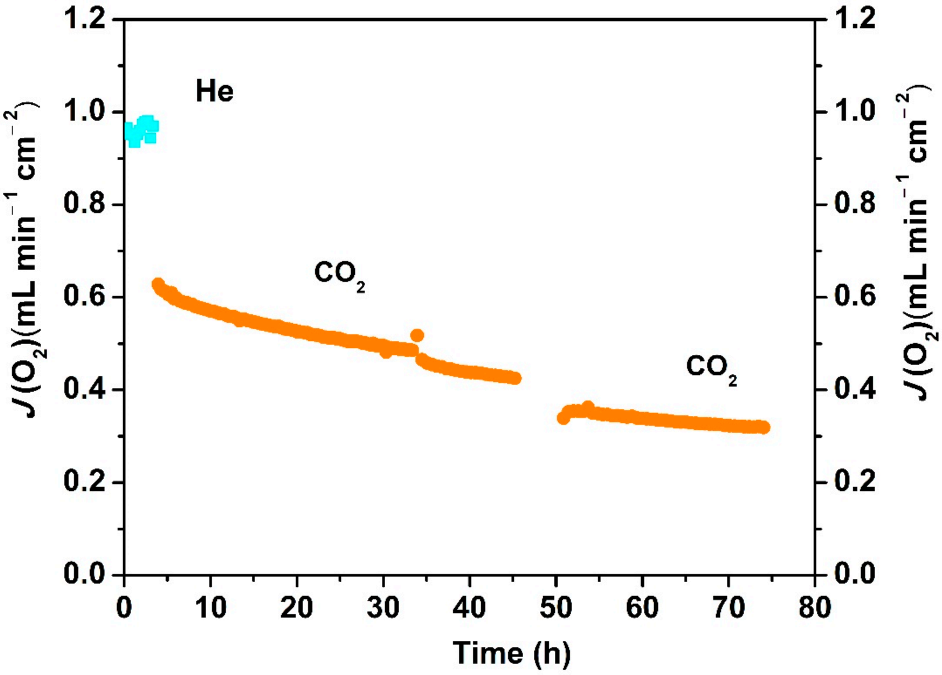

Figure S2. Long-term oxygen permeation flux through a $Ce_{0.9}Pr_{0.1}O_{2-\delta}$–$Nd_{0.5}Sr_{0.5}Fe_{0.9}Cu_{0.1}O_{3-\delta}$ membrane at 1223 K under air/He or air/CO_2 gradient. Test conditions: 150 mL min^{-1} synthetic air as the feed gas; 29 mL min^{-1} CO_2 as the sweep gas; 1 mL min^{-1} Ne as an internal standard gas; membrane thickness: 0.6 mm.

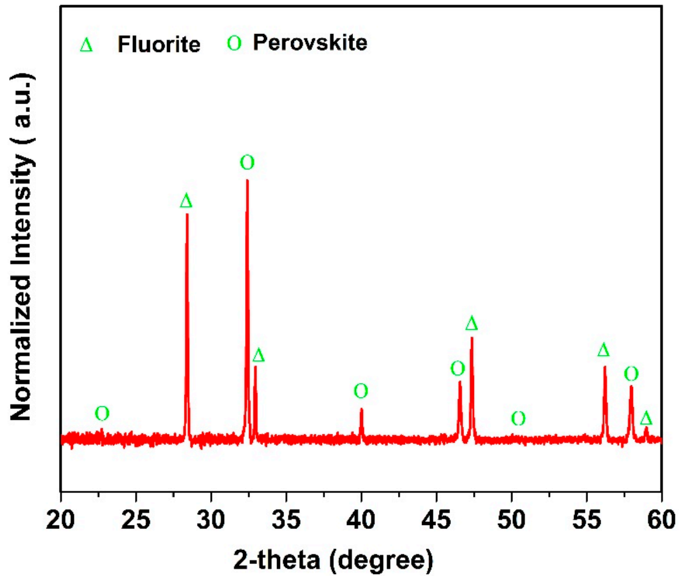

Figure S3. XRD pattern of the sweep side (pure CO_2) of the $Ce_{0.9}Pr_{0.1}O_{2-\delta}$–$Nd_{0.5}Sr_{0.5}Fe_{0.9}Cu_{0.1}O_{3-\delta}$ dual-phase membrane after 70 h O_2 permeation measurements as shown in Figure S2 under air/CO_2 gradient (Bruker D8 Advance instrument with Cu-$K\alpha_{1,2}$ radiation).

Table S1. Summary of the starting material costs generally used in dual-phase membrane materials.

Metal nitrate	Amount	Price	Purity	Euro/ 100 g	Euro/ mol
$Ca(NO_3)_2 \bullet 4H_2O$	500 g	145.0 €	99%	29.0	68.5
$Fe(NO_3)_3 \bullet 9H_2O$	100 g	34.9 €	>98%	34.9	141.0
$Co(NO_3)_3 \bullet 6H_2O$	100 g	88.5 €	>98%	88.5	257.6
$Cu(NO_3)_2 \bullet 2.5H_2O$	100 g	32.1 €	>98%	32.1	74.7
$Sr(NO_3)_2$	100 g	31.8 €	>99%	31.8	67.3
$La(NO_3)_3 \bullet 6H_2O$	100 g	42.6 €	99.9%	42.6	184.5
$Ce(NO_3)_3 \bullet 6H_2O$	100 g	53.5 €	99.5%	53.5	232.3
$Pr(NO_3)_3 \bullet 6H_2O$	250 g	437.0 €	99.9%	174.8	760.4
$Nd(NO_3)_3 \bullet 6H_2O$	100 g	108.0 €	99.9%	108.0	473.4
$Sm(NO_3)_3 \bullet 6H_2O$	100 g	293.0 €	99.9%	293.0	1302.3
$Gd(NO_3)_3 \bullet 6H_2O$	100 g	218.0 €	99.9%	218.0	984.0

The chemicals price is calculated based on the data from Sigma-Aldrich company (www.sigmaaldrich.com) (29/06/2020).

© 2020 by the authors. Submitted for possible open access publication under the terms and conditions of the Creative Commons Attribution (CC BY) license (http://creativecommons.org/licenses/by/4.0/).
